# Factors affecting adolescents’ participation in randomized controlled trials evaluating the effectiveness of healthcare interventions: the case of the STEPSTONES project

**DOI:** 10.1186/s12874-020-01088-7

**Published:** 2020-08-03

**Authors:** Markus Saarijärvi, Lars Wallin, Philip Moons, Hanna Gyllensten, Ewa-Lena Bratt

**Affiliations:** 1 Institute of Health and Care Sciences, University of Gothenburg, Gothenburg, Sweden; 2 Department of Public Health and Primary Care, KU Leuven, Leuven, Belgium; 3 School of Education, Health and Social Studies, Dalarna University, Falun, Sweden; 4 Department of Paediatrics and Child Health, University of Cape Town, Cape Town, South Africa; 5 University of Gothenburg Centre for Person-Centred Care (GPCC), Sahlgrenska Academy, University of Gothenburg, Gothenburg, Sweden; 6 Department of Pediatric Cardiology, The Queen Silvia Children’s Hospital, Gothenburg, Sweden

**Keywords:** Adolescent, Chronic disease, Mixed method, Process evaluation, Randomized controlled trial, Transition of care

## Abstract

**Background:**

Recruitment of adolescents to intervention studies is a known challenge. For randomized controlled trials (RCT) to be generalizable, reach must be assessed, which means ascertaining how many of the intended population actually participated in the trial. The aim of this study was to evaluate the reach and representativeness of an RCT evaluating the effectiveness of a complex intervention for adolescents with chronic conditions.

**Methods:**

A mixed methods sequential explanatory design was employed. Firstly, quantitative cross-sectional data from the RCT, patient registries and medical records were collected and analysed regarding baseline differences between participants and non-participants in the trial. Secondly, qualitative data on their reasons for participating or not were collected and analysed with content analysis to explain the quantitative findings.

**Results:**

Participants showed larger differences in effect sizes and a significantly more complex chronic condition than non-participants. No other statistically significant differences were reported, and effect sizes were negligible. Reasons for declining or accepting participation were categorized into three main categories: altruistic reasons, personal reasons and external reasons and factors.

**Conclusions:**

Integration of quantitative and qualitative findings showed that participation in the RCT was affected by disease complexity, the perceived need to give back to healthcare and research and the adolescents’ willingness to engage in their illness. To empower adolescents with chronic conditions and motivate them to participate in research, future intervention studies should consider developing tailored recruitment strategies and communications with sub-groups that are harder to reach.

## Background

Recruitment of adolescents and young persons with chronic conditions to research studies is a known challenge [1]. For intervention studies, such as randomized controlled trials (RCT), recruitment rates in this population have ranged from 10 to 50% [2–6]. Factors considered to affect adolescents’ participation in RCTs studies may be clinical (e.g. disease severity) [7], demographic (e.g. household income and geographic location), and individual (e.g. personal incentives, trust in healthcare providers and school performance) [8–12]. Moreover, an additional barrier to recruitment is that participation in research studies means the adolescent has to engage in their illness [13]. For many adolescents this is challenging, as young persons want to live their lives as a healthy normal teen, and not spend excessive time thinking about their illness [14].

In most settings, adolescents living with a chronic condition have to transfer their care from a pediatric to an adult setting at age 18. During this transitional phase, these individuals are subject to several risks, such as care gaps in medical follow-up [15], increased healthcare utilization [16, 17], and difficulties in achieving educational and vocational milestones [18]. To prepare adolescents for the transfer to adult care and transition to adulthood, transition programs are advocated [19–21].

RCTs have been conducted to evaluate the effectiveness of transition programs, with positive effects in disease-related knowledge, self-efficacy, self-management [22] and reduced delay in transfer to adult care [23]. However, RCTs evaluating these programs have lacked reporting of components to assess external validity such as representativeness of their samples and response rates [22, 24], so generalizability of current transition programs is limited.

In order to understand how representative an RCT is of its underlying population, and how well findings can be translated into practice, the ‘reach’ is important to consider [25]. Determining the reach of an RCT provides knowledge of the absolute number, proportion and representativeness of individuals who were willing to participate in the trial [26]. When evaluating representativeness of such trials, effectiveness data is rarely enough to understand if the RCT was successful in achieving the outcome. Indeed, RCTs are viewed as the gold standard when evaluating new healthcare innovations such as transition programs [22, 27, 28]. However, RCTs are vulnerable to selection bias. In trials for patients with chronic conditions, as many as 70% of studies including patients with chronic conditions have been found to have samples unrepresentative of the target population [29], with participants often healthier than patients seen in clinical practice [29, 30]. The lack of knowledge on reach and representativeness of participants in transition programs [22, 24] poses several challenges. Firstly, if samples of RCTs do not represent patients in real life settings, clinicians cannot assess which findings that can be translated into clinical practice [31]. Secondly, RCTs are usually the basis for health economic evaluations and decisions on resource allocation in healthcare [32, 33]. If samples of these trials are biased, decision-makers risk making faulty assessments as to which interventions that are cost-effective and could be implemented into practice. Thirdly, we lack knowledge about which adolescents and sub-groups that are more difficult to recruit for RCTs. The aim of this study was therefore to evaluate the reach and representativeness of an RCT evaluating the effectiveness of a healthcare intervention for adolescents with chronic conditions in transition to adulthood. Two specific objectives were formulated to achieve this aim: (i) to compare clinical and demographic characteristics of participants and non-participants in the RCT, and (ii) to describe adolescents’ reasons for participating or not participating in the RCT from their own perspective.

## Methods

### Design

A mixed methods sequential explanatory design was utilized, consisting of two phases [34]. Phase one consisted of quantitative cross-sectional data from the STEPSTONES (Swedish Transition Effects Projects Supporting Teenagers with chrONic mEdical conditionS) project, where a transition program for adolescents with congenital heart disease (CHD) was evaluated through an RCT [35]. Phase two consisted of qualitative data on participants’ and non-participants’ reasons for accepting or declining participation. A mixed methods approach to answering the research question was used to facilitate a deeper understanding of what factors affect reach in complex interventions from a quantitative and qualitative perspective. The Good Reporting of A Mixed Methods Study (GRAMMS) checklist [36] was followed for this article (additional file 1). The present study is part of a larger process evaluation study of the STEPSTONES project in which the implementation, mechanisms of impact and contextual factors are explored within the RCT [37]. A study protocol describing the full extent of the process evaluation has been previously published [38].

### Setting

In Sweden, care for individuals living with CHD is mainly organized around seven university hospitals (CHD-centres). However, follow-up is also provided at regional hospitals connected to the university hospitals. These seven centres provide specialized care for both pediatric and adult patients with CHD, with the transfer to adult care occurring at age 18. Medical follow up is based on current treatment guidelines with the follow-up frequency being determined by the complexity of the CHD, varying between visits on a yearly basis to every 3–5 years [39].

#### The intervention study

**Fig. 1:**
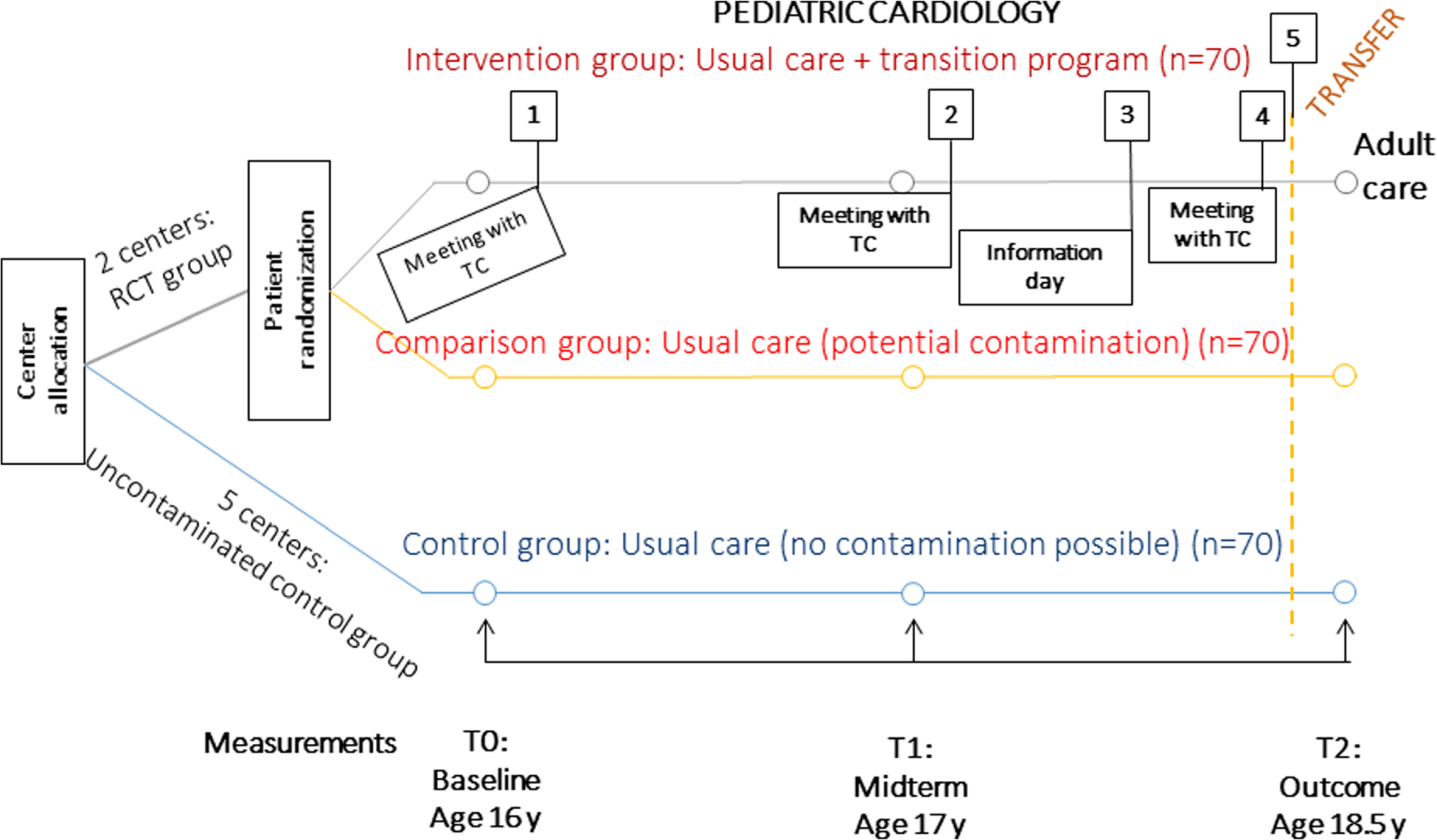
Flow chart of STEPSTONES RCT. TC = Transition Coordinator

The STEPSTONES project was performed at the seven CHD centres in Sweden, with the hypothesis that adolescents (age 16–18.5 years) participating in the transition program in addition to usual care will have a higher patient empowerment score than adolescents receiving only usual care (primary outcome) [40]. The transition program is a complex intervention comprising multiple components delivered by a transition coordinator who works at the outpatient pediatric cardiology clinic. In short, the transition program entails three outpatient visits with 1-year intervals and one information day for adolescents and their parents [35]. The effects of the transition program are evaluated using a hybrid RCT where a longitudinal observational study is embedded within a conventional RCT (see Fig. 1). The two largest centres are designated as RCT centres, where participants are individually randomized to either the intervention group or the comparison group. The remaining five centres are control groups in which no contamination from the intervention is possible. The total sample size of the trial is 210 patients with 70 patients in each arm, based on power calculation of the primary outcome [35]. For the present study, only the RCT centres were included in the analysis.

### Sample

**Fig. 2:**
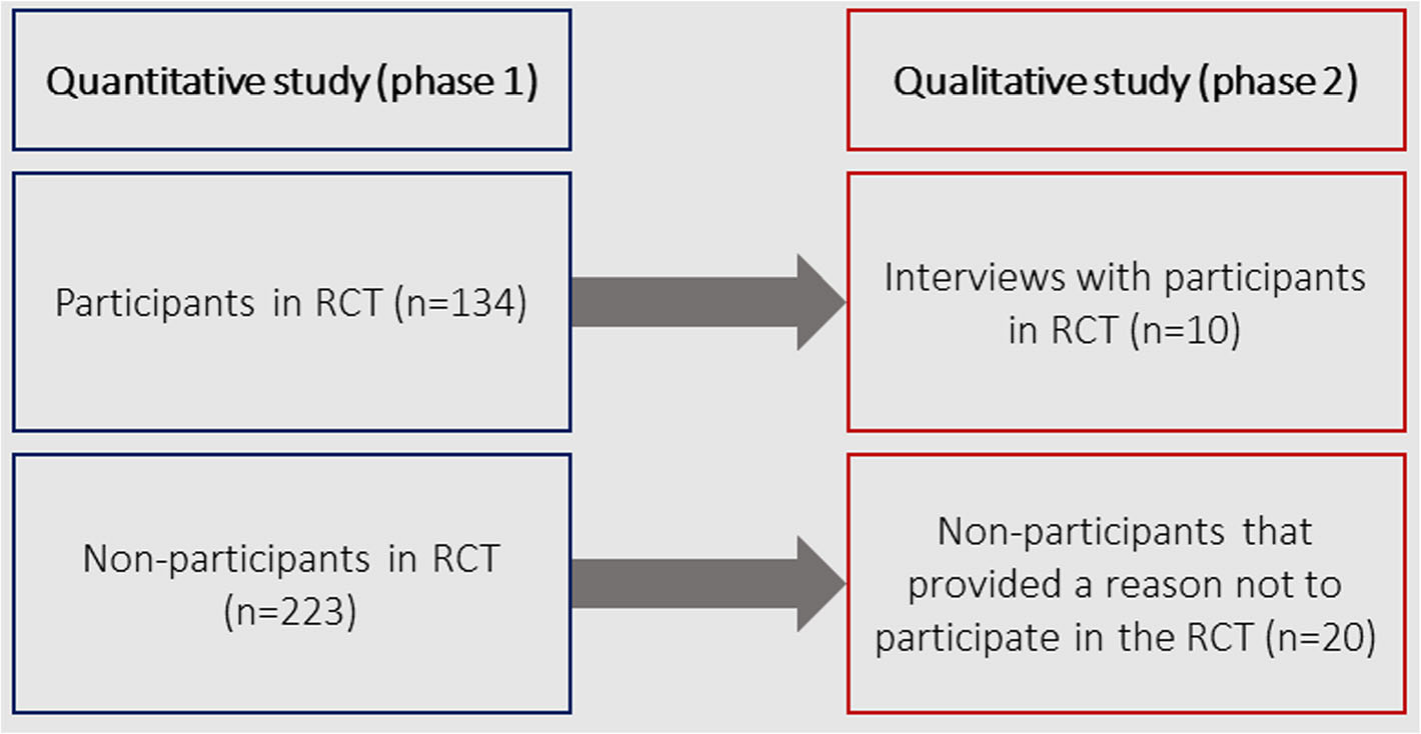
Flow chart of quantitative and qualitative phases in the study

The inclusion criteria for the present study were the same as those of the RCT: 1) age 16 at recruitment, 2) literate, 3) Swedish-speaking, and 4) having a CHD as defined by Mitchell et al. [41] with follow-up planned in an Adult Congenital Heart Disease (ACHD) clinic. Exclusion criteria were: 1) previous heart transplantation, and 2) illiterate or non-Swedish speaking. The cohort for the quantitative study (phase 1) was categorized into two groups: participants (*n* = 134) and non-participants (*n* = 223). Participants were patients who had consented to participate in the study and had been randomized to either intervention or control group. Non-participants were patients who either actively declined participation, were unreachable, or did not return the informed consent to participate in the study despite several reminders. For the qualitative study (phase 2), we used nested sampling whereby a sub-sample of patients from the group of participants (*n* = 10) and non-participants (*n* = 20) were sampled for participation [34] (see Fig. 2).

### Data collection

Quantitative data was retrieved from the national register SWEDCON (SWEDish register of CONgential heart disease) (http://www.ucr.uu.se/swedcon.se) [42] and medical records. Patients’ medical records were screened for residential address and Google Maps™ was used to calculate the geographical distance between the patients’ home and the hospital where the transition program (i.e. intervention) was delivered.

Qualitative data were collected from two sources. All patients declining participation in the STEPSTONES project at the time of recruitment to the RCT were asked their reasons for doing so by the data collection officer. If the patient provided a reason, this was documented in the RCT enrolment forms. For patients accepting participation in the RCT, data were collected from patients participating in the intervention group after participation in the RCT through semi-structured interviews. In total, 10 interviews were carried out with adolescents aged 18–19 by the main author (MS). In the sampling of participants, we employed purposive sampling to select a diverse group with maximum variation [43] in terms of disease complexity, sex and intervention centre. The interviews were carried out face-to-face (*n* = 1), over the telephone (*n* = 5) or email (*n* = 4) as we wanted the adolescents to choose the communication medium that they were most comfortable with [44, 45]. The interviews comprised three questions: *“Can you tell me why you agreed to participate in the study*?”, *“What do you think are the benefits of participating in this kind of study?”* and *“What do you think are reasons for not wanting to participate in this kind of study?”,* followed by probing questions (additional file 2). Due to constrictions in the ethical approval received for this study, non-participants could not be approached for probing questions. Therefore, in the interviews performed with the participants, we added the third question on reasons for not wanting to participate in this kind of study in order to illuminate and give more depth to potential reasons on not wanting to participate from the adolescents’ point-of-view. The interviews performed face-to-face or over the telephone were audio-recorded and transcribed verbatim.

### Data analysis

#### Quantitative data

Statistical analyses were performed in SPSS (Statistical Package for Social Sciences) v.24**.** As the continuous variables were not normally distributed, they were represented as medians and interquartile ranges. Categorical variables were expressed as absolute numbers and proportions. Variables concerning clinical, demographic and health service use were compared between participants and non-participants using significance testing (significance level < 0.05). The chi-square test and Fishers’ exact test was used for categorical variables and the Mann-Whitney U test for continuous variables. In order to calculate the magnitude of difference between participants and non-participants, we calculated effect sizes. For categorical variables, we used Cohens’ *w*. For continuous variables, we used the Wilcoxon’s test by calculating *r* = Z/ √*n.* Z represents the Wilcoxon test statistic, which is the Mann-Whitney U Z statistic. Cut-offs for Cohens *w* and *r* were graded as follows: 0.1–0.3 = small difference, 0.3–0.5 = moderate difference, and > 0.5 = large difference [46].

#### Qualitative data

Inductive content analysis of the manifest content of reasons for participation and non-participation was performed according to Graneheim and Lundman [47]. This analysis method is suitable for analysing texts and was therefore appropriate due to our using data from various sources. The analysis was performed with the computer-assisted qualitative analysis software, NVivo version 12. We chose to analyse and present the data from the participants and non-participants as a whole. This was because the texts from the RCT recruitment protocols (non-participants’ reasons) where short summaries, and the interviews (participants) provided more depth and explanations that were not present in the summaries from the recruitment protocols. The text from the recruitment protocols and transcribed interviews were read through several times to get a grasp of the whole. Meaning units were then identified as constellations of words and sentences that answered the aim of the study and contained a common meaning. These meaning units were then condensed, which in some cases meant a reducing the number of words but keeping the core essence of the meaning unit. In this process, codes were created and compared to each other, with those that were similar in content sorted into the same group. These groups formed the basis for subcategories. This procedure was repeated for the subcategories, resulting in the main categories [47]. Two of the authors (MS & ELB) with experience in qualitative methods and content analysis were responsible for carrying out the analysis and continuously discussed the findings with the other co-authors.

### Ethical considerations

The study was approved by the Regional Ethical Review Board in Gothenburg, Sweden (No.931–15) and the board of directors of SWEDCON. The study was performed in accordance with the Declaration of Helsinki [48]. For the quantitative study, data was retrieved from a national register, so informed consent could not be retrieved from each individual participant for this particular study. However, each patient enrolled in the register is informed that their data could be used for research purposes. In addition, the patient is informed about their right to refuse permission to use their data. For the qualitative study, informed consent was retrieved from participants after they had been given oral and written information about the study. Non-participants who provided a reason for declining were informed that their answers would be used for research purposes and consented to this. All data were stored coded and password-protected. During the interview period, the interviews performed over email were copied to a password-protected database and stored in a designated folder in the main authors email program. When the analysis was completed, the emails were deleted from the email program to protect the participants’ confidentiality.

## Results

### Phase 1 – quantitative study

From the total eligible population identified in SWEDCON (*n* = 357), 37.5% (*n* = 134) participated in the STEPSTONES RCT. Statistically significant differences were observed between participants and non-participants for primary diagnosis and disease complexity (Table 1). A larger proportion of participants had a more complex CHD than non-participants. The statistically significant results also presented large effect sizes for primary diagnosis w = 1.46 and disease complexity w = 0.54. There were not statistical differences between participants and non-participants for sex, geographic distance to hospital, proportion receiving cardiac pharmacotherapy, proportion receiving special aid in school, and the number of cardiac interventions and surgeries, and the effect sizes were negligible.

**Table 1:** Demographic and clinical characteristics of participants and non-participants

Quantitative study						Qualitative study	
	Participants *n* = 134 (%)	Non-participants *n* = 223 (%)	Statistical test	*p*-value	Effect size*	Participants *n* = 10 (%)	Non-participants *n* = 20 (%)
Female sex	61 (45.5)	99 (44.4)	*X*^2^ = 0.43	*p* = 0.836	w = 0.02	5 (50)	10 (50)
Distance to hospital (km)	19.9 (IQR = 20.8)	16.5 (IQR = 20.4)	Mann-Whitney *U* = 14,002 *z* = −0.65	*p* = 0.516	r = − 0.03	–	–
Proportion with cardiac pharmacotherapy	18 (13.4)	34 (16)	*X*^2^ = 0.344	*p* = 0.557	w = 0.02	–	–
Proportion with special aid in school	5 (4)	13 (5.8)	*X*^2^ = 1.032	*p* = 0.31	w = 0.06	–	–
Disease complexity**			*X*^2^ = 10.195	*p* = 0.017	w = 0.54		
Mild	14 (10.4)	52 (23.3)				3 (30)	3 (15)
Moderate	84 (62.7)	119 (53.4)				6 (60)	12 (60)
Severe	35 (26.2)	48 (21.5)				1 (10)	5 (25)
Primary diagnosis			Fishers’ exact test = 27.634	*p* = 0.023	w = 1.46	–	–
Single ventricle physiology	5 (3.7)	10 (4.5)					
Tricuspid valve abnormalities	1 (0.7)	4 (1.8)					
Tetralogy of Fallot	15 (11.1)	16 (7.2)					
Double outlet right ventricle	0 (0.0)	10 (4.5)					
Truncus arteriosus	1 (0.7)	2 (0.9)					
Transposition of the great arteries	20 (14.9)	15 (6.7)					
Coarctation of the aorta	25 (18.7)	26 (11.7)					
Atrioventricular septal defect	4 (3.0)	5 (2.2)					
Ebstein anomaly	1 (0.7)	2 (0.9)					
Pulmonary valve abnormalities	15 (11.2)	22 (9.9)					
Aortic valve abnormalities	26 (19.4)	37 (16.6)					
Atrial septal defect	5 (3.7)	16 (7.2)					
Ventricular septal defect	9 (6.7)	30 (13.5)					
Mitral valve abnormalities	3 (2.2)	13 (5.8)					
Pulmonary vein abnormalities	3 (2.2)	9 (4.0)					
Patent ductus arteriosus	1 (0.7)	4 (1.8)					
Other	0 (0.0)	1 (0.4)					
Number of cardiac operations	1.0 (IQR = 2)	1.0 (IQR = 2)	Mann-Whitney *U* = 13,899.500 *z* = −1.148	*p* = 0.251	r = −0.06	–	–
Number of catheterizations	0.00 (IQR = 0)	0.00 (IQR = 0)	Mann-Whitney *U*= 14,402 *z* = − 1.08	*p* = 0.28	r = −0.05	–	–

*Cut-offs for Cohen’s w and Cohen’s r = 0.1–0.3 small; 0.3–0.5 = moderate; > 0.5 = large

**Complexity of CHD categorized according to 2018 AHA/ACC Guideline for Management of Adults with Congenital Heart Disease

### Phase 2 – qualitative study

Table 1 presents the demographic characteristics of the participants in the qualitative study (*n* = 30). The analysis resulted in three main categories, with adjacent subcategories (Table 2).

**Table 2:** Categories and subcategories from the qualitative content analysis

	Altruistic reasons	Personal reasons	External reasons and factors
***Reasons for participating***	*To help other adolescents in the same situation* *To contribute to research and improved care* *Participating was the right thing to do*	*Meeting others in the same situation* *Educational objectives*	*Parents as facilitators*
***Reasons for not participating***		*Meeting others in the same situation* *Being healthy and therefore not needing the study* *Fearing what the study would evoke and demand of me*	*Parents as barriers*

### Altruistic reasons

In this category, reasons that facilitated participation were addressed. This category was based on three subcategories: helping other adolescents in the same situation, contributing to research and improved care, and participating was the right thing to do.

#### Helping other adolescents in the same situation

This subcategory included the wish to help other adolescents in the same situation, even if their participation did not help themselves. Believed reasons were that they could be supportive by showing other adolescents in the same situation that they were not alone and that other adolescents were going through the same process of transitioning into adulthood.

*“Yes, for example, I think mentally they know they are not alone; there are other people who have similar problems and who behave similarly in life. And maybe ... many have different ways of acting ... but I think it helps people to show that they are not alone…”*

- *19-year-old (ID: 2030).*

#### Contributing to research and improved care

Here, the participants described a wish to contribute to research by sharing their experiences, but also through their participation in the study.

*“Why I did was because I felt kind of like I might as well help, I think it’s good that you carry out studies and research like this and then I thought in that case, I might as well because I’m experiencing this – I can share my experiences. And how I felt about this whole process and all that.”*

- *19-year-old (ID: 2047).*

#### Participating was the right thing to do

For some participants, this subcategory was shaped on the assumption that participation in a research study was a good deed and that deciding to contribute came naturally.

### Personal reasons

This category highlighted personal reasons for participating or not participating in the study. Four subcategories were identified that were considered factors affecting the decision to participate.

#### Meeting others in the same situation

Being given the opportunity to meet other adolescents in the same situation could lead to participants and others in the same situation feeling less alone. Another aspect was the need to show others that there are young people with CHD who are doing well and living normal lives.

*“Yes exactly! Seeing that you are not alone is something that very often satisfies the brain.”*

- *19-year-old (ID: 2030).*

*“…that there are people who are also doing well with this heart disease…”*

- *19-year-old (ID: 2030).*

However, meeting other young adolescents with CHD could be a reason for not wanting to participate, as some adolescents felt it was awkward to talk about their condition in front of others.

#### Educational objectives

Wanting to learn more about CHD and how the condition would affect the adolescents in their daily lives were reasons that facilitated participation. The adolescents considered participating in the study an advantage to participate in the study because they saw it as an opportunity to learn more about their illness, the transfer to adult care and the transition to adulthood with a chronic condition.

*“It’s also clear that there were benefits from this study for me as well; it gave back to me a bit because the study meant a lot that I would learn about my illness - then I understood”*

- *19-year-old (ID: 1051).*

*“Well... maybe I wanted more understanding of what it was because I didn't even know ... I’d just heard that I had something with my heart and I kind of went to the doctor sometimes. I didn’t know what it was at all. But then maybe I want more information about it.”*

- *19-year-old (ID: 2038).*

#### Feeling healthy and therefore not needing the study

This subcategory described why adolescents did not participate in the study due to not feeling ill or affected by their CHD. For this reason, they also believed they would be a bad fit for the study. Moreover, there as a perception among the adolescents that young people participating in studies like this were affected more severely by CHD than they were.

*“And I also think that there are probably a lot of people who aren’t really affected by their CHD, just like me, and then maybe they feel that there’s even less reason for them to participate. Like that you don’t fit in there in any way, that you have nothing to do with the study because you are not characterized by your heart failure.”*

- *19-year-old (ID: 1051).*

#### Fearing what the study would evoke and demand of them

This subcategory comprised both emotional and practical reasons for adolescents choosing not to participate. Most commonly, adolescents expressed a perception that youths with CHD were afraid to think and talk about their health, and were fed up with having a chronic illness.

*“Like…if you’ve gone through a lot of surgeries and examinations….that it’s a big part of their life. It’s not that much fun you know. We met a guy who’d gone through at least 8 operations in his life. So it might be sensitive to talk about that. You never know what’s gonna happen and if you survive….I don’t know, it might be difficult. It’s not something that you go round bragging about”*

- *18-year-old (ID: 2080).*

Practical reasons were also given for not participating, such as not having enough time to contribute to the study, that the hospital was too far away from the patient’s home, and that the additional hospital visits that randomization to the intervention group would entail would be emotionally and practically stressful for the adolescents.

### External reasons and factors

The final category included reasons and factors that were external to the adolescents’ own perceptions and decisions to participate in the research study. Here, parental influence on the decision to participate was illustrated in two subcategories.

#### Parents as facilitators

Parents could be active partners in the decision to participate in the study.

*“It was kind of a collective decision by both my parents; if you are 16, you are usually a little indecisive, very hard to think that you shouldn’t do it. But we talked it over briefly and thought like, why not? So nothing wrong with that. So you could say it was a bit of a joint decision, but it was a decision that I would also like to make myself, you could say.”*

- *19-year-old (ID: 2030).*

#### Parents as barriers

In this subcategory, there were reasons as to why parents could potentially be barriers to participation. Domestic issues, such as conflicts within the family, were also brought up as a barrier to participation.

*“Maybe that parents are more involved and tell the kids that they should not join, that it is unnecessary or so”*

- *19-year-old (ID: 2047).*

## Discussion

Having integrated findings from the quantitative and qualitative phases of this study, we can conclude that participants differed from non-participants in regard to disease complexity. A potential reason for this may be that adolescents with a more complex conditions felt grateful towards research and the healthcare system, and were thus motivated to give back. In contrast, non-participants (who as a group generally had a less complex condition) mentioned not wanting to engage in their illness more than necessary and not recognizing the illness as a part of their social identity as reasons for declining participation. These findings are in line with results from previous studies in this patient population [49]. It is also in keeping with observations in other patient populations, for instance, in RCTs concerning young persons with asthma, where non-participants have been shown to be healthier and less likely to require medication [2]. Differences in quality of life and health status could also be a reason why patients with more severe conditions want to participate in a transition intervention. Several studies have shown that patients with moderate and severe complexity of CHD have a lower health related quality of life than patients with milder CHD [50–52]. Moreover, adolescents with complex CHD experience lower self-reported health and physical functioning [53, 54]. Both health status and quality of life might be incentives that affect participation in an RCT evaluating an intervention aiming to improve the adolescents’ ability to manage their health and care in adulthood.

An important aspect to consider regarding representativeness of the sample of this study is that patients with complex CHD generally have a higher frequency of outpatient visits [39]. As patients in the STEPSTONES RCT had a more complex CHD than those who declined participation, this will have an impact on upcoming economic evaluations, as patients seen in the RCT are the primary data source for cost-effectiveness ratios. It is therefore important for upcoming studies evaluating similar interventions to consider performing budget impact analyses adjacent to cost effectiveness studies in order to estimate the true healthcare budget impact of complex healthcare interventions [55]. In the present study, we did not include a variable on health service use as SWEDCON was established in 2009 and data for this variable has not been added retrospectively for all centres. As young persons with CHD consume more health care resources during the early years of life [56], including this variable would have introduced bias into our results.

Although our findings suggest that disease complexity may be one factor affecting participation in an RCT evaluating a complex healthcare intervention for adolescents, our results should be interpreted bearing in mind some methodological caveats. Firstly, we did not have access to individual level data on socioeconomic status for the complete sample. This has been an important factor in previous studies comparing participants to non-participants, where poor socioeconomic status has been a barrier to participation [7–10]. Indeed, domestic issues were also raised in our qualitative findings as a potential reason for declining participation. Secondly, we did not have any measure of self-reported health. The health status measure registered in SWEDCON, PedsQL 4.0 [57], has been recently implemented into standard practice in pediatric cardiology and coverage in the register is therefore limited. Thirdly, as multiple comparisons between groups increases the risk for type I errors we also used the effect size estimates. The effect size is independent of sample size and significance level, and measure the extent of an association between variables which is a more accurate basis for treatment decisions [58, 59]. Finally, we only received reasons for declining participation from 8% of all non-participants, which makes it difficult to generalize the findings. Furthermore, as ethical constrictions prevented us from interviewing these non-participants, we had to rely on the short statements they had provided on the RCT enrolment forms.

In the light of these limitations, this study has several strengths. To our knowledge, this is the first published study assessing the reach of an RCT evaluating a transition program for adolescents with chronic condition. As evidence on transition programs is scarce [60], this is an important step in providing evidence on effectiveness. The recruitment for the RCT was based on a register which has high concordance with the medical records, therefore increasing validity [61]. Moreover, by reporting data on the reach and representativeness of this RCT before the outcomes of the effectiveness evaluation, we can avoid making biased interpretation of outcomes [37]. Finally, the use of mixed methods, where qualitative methods were used to explain quantitative findings, gave a deeper understanding of factors that impact on the decision to participate or not in intervention studies for young persons living with chronic conditions. One insight especially gained from mixing methods was that adolescents with a milder condition might benefit from tailored messages while being recruited to intervention studies. From our findings, these patients are more likely to decline participation due to, for example, not wanting to engage in their illness and fear of standing out for the wrong reasons. In future trials with this patient population, researchers and clinicians should carefully consider to this issue. Indeed, it would be useful to involve young persons in the development phase of the intervention, and then not only focus on developing the clinical intervention but also focus on effective recruitment strategies with tailored messages that can empower adolescents with milder conditions to participate in research trials [62, 63].

## Conclusion

Factors positively affecting adolescents’ participation in RCTs evaluating healthcare interventions are higher disease complexity and a will to give back to research and healthcare, along with an interest to learn more about their health and care. Factors that negatively affect participation negatively are milder disease complexity, along with the feeling of being healthy and not wanting to engage in the illness. Future studies within this area should investigate the impact of socio-demographic variables on study participation, along with self-reported health. The creation of tailored recruitment messages with relevant stakeholders to reach hard-to-recruit sub-groups is an area which needs increased attention and a topic for future research initiatives.

## Supplementary information

**Additional file 1.** Good Reporting of A Mixed Methods Study (GRAMMS) Checklist.

**Additional file 2.** Interview guide for the qualitative study.

## Data Availability

All data and materials are available from the corresponding author upon request.

## Abbreviations

RCT: Randomized Controlled Trial
STEPSTONES: Swedish Transition Effects Project Supporting Teenagers with chrONic mEdical conditionS
GRAMMS: Good Reporting of A Mixed Methods Study checklist
CHD: Congenital Heart Disease
ACHD: Adult Congenital Heart Disease
SWEDCON: SWEDish CONgenital heart disease register
SPSS: Statistical Package for Social Sciences
PedsQL 4.0: Pediatric Quality of Life Inventory

## References

[1] Nguyen TT, Jayadeva V, Cizza G, Brown RJ, Nandagopal R, Rodriguez LM, Rother KI (2014). Challenging recruitment of youth with type 2 diabetes into clinical trials. J Adolesc Health.

[2] Joseph CL, Saltzgaber J, Havstad SL, Johnson CC, Johnson D, Peterson EL, Alexander G, Couper MP, Ownby DR (2011). Comparison of early-, late-, and non-participants in a school-based asthma management program for urban high school students. Trials..

[3] Crutzen R, Bosma H, Havas J, Feron F. What can we learn from a failed trial: insight into non-participation in a chat-based intervention trial for adolescents with psychosocial problems. BMC Res Notes. 2014; 20;7:824. 10.1186/1756-0500-7-824 PMC4247599 25409911

[4] Oh AY, Davis T, Dwyer LA, Hennessy E, Li T, Yaroch AL, Nebeling LC (2017). Recruitment, enrollment, and response of parent-adolescent dyads in the FLASHE study. Am J Prev Med.

[5] Hendricks-Ferguson VL, Cherven BO, Burns DS, Docherty SL, Phillips-Salimi CR, Roll L, Stegenga KA, Donovan Stickler M, Haase JE (2013). Recruitment strategies and rates of a multi-site behavioral intervention for adolescents and young adults with cancer. J Pediatr Health Care.

[6] Loban A, Mandefield L, Hind D, Bradburn M (2017). A randomized trial found online questionnaires supplemented by postal reminders generated a cost-effective and generalizable sample but don't forget the reminders. J Clin Epidemiol.

[7] Sanford SD, Beaumont JL, Snyder MA, Reichek J, Salsman JM (2017). Clinical research participation among adolescent and young adults at an NCI-designated Comprehensive Cancer Center and affiliated pediatric hospital. Support Care Cancer.

[8] Roick J, Danker H, Kersting A, Briest S, Dietrich A, Dietz A, Einenkel J, Papsdorf K, Lordick F, Meixensberger J, Mossner J, Niederwieser D, Prietzel T, Schiefke F, Stolzenburg JU, Wirtz H, Singer S. Factors associated with non-participation and dropout among cancer patients in a cluster-randomised controlled trial. Eur J Cancer Care. 2018;27(1). 10.1111/ecc.12645 28134477

[9] Winding TN, Andersen JH, Labriola M, Nohr EA (2014). Initial non-participation and loss to follow-up in a Danish youth cohort: implications for relative risk estimates. J Epidemiol Commun Health.

[10] Perez RG, Ezpeleta L, Domenech JM (2007). Features associated with the non-participation and drop out by socially-at-risk children and adolescents in mental-health epidemiological studies. Soc Psychiatry Psychiatr Epidemiol.

[11] van Dijk-Lokkart EM, Braam KI, Huisman J, Kaspers GJ, Takken T, Veening MA, Bierings M, Merks JH, Grootenhuis MA, van den Heuvel-Eibrink M, Streng IC, van Dulmen-den Broeder E (2015). Factors influencing childhood cancer patients to participate in a combined physical and psychosocial intervention program: quality of life in motion. Psycho-Oncol.

[12] Crane S, Broome ME (2017). Understanding ethical issues of research participation from the perspective of participating children and adolescents: a systematic review. Worldviews Evid-Based Nurs.

[13] Hudson BF, Oostendorp LJ, Candy B, Vickerstaff V, Jones L, Lakhanpaul M, Bluebond-Langner M, Stone P (2017). The under reporting of recruitment strategies in research with children with life-threatening illnesses: a systematic review. Palliat Med.

[14] Chong LSH, Fitzgerald DA, Craig JC, Manera KE, Hanson CS, Celermajer D, Ayer J, Kasparian NA, Tong A (2018). Children’s experiences of congenital heart disease: a systematic review of qualitative studies. Eur J Pediatr.

[15] Heery E, Sheehan AM, While AE, Coyne I (2015). Experiences and outcomes of transition from pediatric to adult health Care Services for Young People with congenital heart disease: a systematic review. Congenit Heart Dis.

[16] Burchill LJ, Gao L, Kovacs AH, Opotowsky AR, Maxwell BG, Minnier J, Khan AM, Broberg CS (2018). Hospitalization trends and health resource use for adult congenital heart disease-related heart failure. J Am Heart Assoc.

[17] Singh S, Desai R, Fong HK, Sadolikar A, Samani S, Goyal H (2018). Extra-cardiac comorbidities or complications in adults with congenital heart disease: a nationwide inpatient experience in the United States. Cardiovasc Diagnosis Ther.

[18] Stam H, Hartman EE, Deurloo JA, Groothoff J, Grootenhuis MA (2006). Young adult patients with a history of pediatric disease: impact on course of life and transition into adulthood. J Adolesc Health.

[19] Mazur A, Dembinski L, Schrier L, Hadjipanayis A, Michaud PA (2017). European academy of Paediatric consensus statement on successful transition from paediatric to adult care for adolescents with chronic conditions. Acta Paediatr.

[20] American Academy of Pediatrics, American Academy of Family Physicians, American College of Physicians-American Society of Internal Medicine (2002). A consensus statement on health care transitions for young adults with special health care needs. Pediatrics.

[21] Sable C, Foster E, Uzark K, Bjornsen K, Canobbio MM, Connolly HM, Graham TP, Gurvitz MZ, Kovacs A, Meadows AK, Reid GJ, Reiss JG, Rosenbaum KN, Sagerman PJ, Saidi A, Schonberg R, Shah S, Tong E, Williams RG (2011). Best practices in managing transition to adulthood for adolescents with congenital heart disease: the transition process and medical and psychosocial issues: a scientific statement from the American Heart Association. Circulation..

[22] Campbell F, Biggs K, Aldiss SK, O'Neill PM, Clowes M, McDonagh J, While A, Gibson F (2016). Transition of care for adolescents from paediatric services to adult health services. Cochrane Database Syst Rev.

[23] Mackie AS, Rempel GR, Kovacs AH, Kaufman M, Rankin KN, Jelen A, Yaskina M, Sananes R, Oechslin E, Dragieva D, Mustafa S, Williams E, Schuh M, Manlhiot C, Anthony SJ, Magill-Evans J, Nicholas D, McCrindle BW (2018). Transition intervention for adolescents with congenital heart disease. J Am Coll Cardiol.

[24] Le Roux E, Mellerio H, Guilmin-Crepon S, Gottot S, Jacquin P, Boulkedid R, Alberti C (2017). Methodology used in comparative studies assessing programmes of transition from paediatrics to adult care programmes: a systematic review. BMJ Open.

[25] Craig P, Dieppe P, Macintyre S, Michie S, Nazareth I, Petticrew M (2013). Developing and evaluating complex interventions: the new Medical Research Council guidance. Int J Nurs Stud.

[26] Glasgow RE, Vogt TM, Boles SM (1999). Evaluating the public health impact of health promotion interventions: the RE-AIM framework. J Public Health.

[27] Sibbald B, Roland M (1998). Understanding controlled trials. Why are randomised controlled trials important?. BMJ.

[28] Schulz KF, Altman DG, Moher D (2010). CONSORT 2010 statement: updated guidelines for reporting parallel group randomized trials. Ann Intern Med.

[29] Kennedy-Martin T, Curtis S, Faries D, Robinson S, Johnston J (2015). A literature review on the representativeness of randomized controlled trial samples and implications for the external validity of trial results. Trials..

[30] de Boer SP, Lenzen MJ, Oemrawsingh RM, Simsek C, Duckers HJ, van der Giessen WJ, Serruys PW, Boersma E (2011). Evaluating the ‘all-comers’ design: a comparison of participants in two ‘all-comers’ PCI trials with non-participants. Eur Heart J.

[31] Ferguson L (2004). External validity, generalizability, and knowledge utilization. J Nurs Scholarsh.

[32] Baltussen R, Leidl R, Ament A (1999). Real world designs in economic evaluation. Bridging the gap between clinical research and policy-making. Pharmacoeconomics..

[33] Ramsey SD, Willke RJ, Glick H, Reed SD, Augustovski F, Jonsson B, Briggs A, Sullivan SD (2015). Cost-effectiveness analysis alongside clinical trials II-an ISPOR good research practices task force report. Value Health.

[34] Creswell JW. Designing and conducting mixed methods research. 2nd ed. Plano Clark VL, editor. Los Angeles: Los Angeles : SAGE Publications; 2011.

[35] Acuña Mora M, Sparud-Lundin C, Bratt E-L, Moons P (2017). Person-centred transition programme to empower adolescents with congenital heart disease in the transition to adulthood: a study protocol for a hybrid randomised controlled trial (STEPSTONES project). BMJ Open.

[36] O'Cathain A, Murphy E, Nicholl J (2008). The quality of mixed methods studies in health services research. J Health Serv Res Policy.

[37] Moore GF, Audrey S, Barker M, Bond L, Bonell C, Hardeman W, Moore L, O'Cathain A, Tinati T, Wight D, Baird J (2015). Process evaluation of complex interventions: Medical Research Council guidance. BMJ (Clin Res ed).

[38] Saarijärvi M, Wallin L, Moons P, Gyllensten H, Bratt E-L (2019). Transition program for adolescents with congenital heart disease in transition to adulthood: protocol for a mixed-method process evaluation study (the STEPSTONES project). BMJ Open.

[39] Stout KK, Daniels CJ, Aboulhosn JA, Bozkurt B, Broberg CS, Colman JM, Crumb SR, Dearani JA, Fuller S, Gurvitz M, Khairy P, Landzberg MJ, Saidi A, Valente AM, Van Hare GF (2018). 2018 AHA/ACC guideline for the Management of Adults with Congenital Heart Disease. Circulation..

[40] Acuna Mora M, Luyckx K, Sparud-Lundin C, Peeters M, van Staa A, Sattoe J, Bratt EL, Moons P (2018). Patient empowerment in young persons with chronic conditions: psychometric properties of the Gothenburg young persons empowerment scale (GYPES). PLoS One.

[41] Mitchell S, Korones S, Berendes H (1971). Congenital heart disease in 56,109 births incidence and natural history. Circulation..

[42] SWEDCON. SWEDCON annual report. http://www.ucr.uu.se/swedcon/arsrapporter2017.

[43] Palinkas LA, Horwitz SM, Green CA, Wisdom JP, Duan N, Hoagwood K (2015). Purposeful sampling for qualitative data collection and analysis in mixed method implementation research. Admin Pol Ment Health.

[44] Hershberger PE, Kavanaugh K (2017). Comparing appropriateness and equivalence of email interviews to phone interviews in qualitative research on reproductive decisions. App Nurs Res.

[45] Neville S, Adams J, Cook C (2016). Using internet-based approaches to collect qualitative data from vulnerable groups: reflections from the field. Contemp Nurse.

[46] Cohen J (1992). A power primer. Psychol Bull.

[47] Graneheim UH, Lundman B (2004). Qualitative content analysis in nursing research: concepts, procedures and measures to achieve trustworthiness. Nurse Educ Today.

[48] World Medical Association. Declaration of Helsinki: ethical principles for medical research involving human subjects. JAMA. 2013 Nov 27; 310 (20):2191–4. 10.1001/jama.2013.281053 24141714

[49] Berghammer MC, Mattsson E, Johansson B, Moons P, Dellborg M (2017). Comparison of participants and non-participants in patient-reported outcome surveys: the case of assessment of patterns of patient-reported outcomes in adults with congenital heart disease–international study. Cardiol Young.

[50] Drakouli M, Petsios K, Giannakopoulou M, Patiraki E, Voutoufianaki I, Matziou V (2015). Determinants of quality of life in children and adolescents with CHD: a systematic review. Cardiol Young.

[51] Mellion K, Uzark K, Cassedy A, Drotar D, Wernovsky G, Newburger JW, Mahony L, Mussatto K, Cohen M, Limbers C, Marino BS (2014). Health-related quality of life outcomes in children and adolescents with congenital heart disease. J Pediatr.

[52] Amedro P, Dorka R, Moniotte S, Guillaumont S, Fraisse A, Kreitmann B, Borm B, Bertet H, Barrea C, Ovaert C, Sluysmans T, De La Villeon G, Vincenti M, Voisin M, Auquier P, Picot MC (2015). Quality of life of children with congenital heart diseases: a multicenter controlled cross-sectional study. Pediatr Cardiol.

[53] Bratt EL, Luyckx K, Goossens E, Budts W, Moons P (2015). Patient-reported health in young people with congenital heart disease transitioning to adulthood. J Adolesc Health.

[54] Kahr PC, Radke RM, Orwat S, Baumgartner H, Diller GP (2015). Analysis of associations between congenital heart defect complexity and health-related quality of life using a meta-analytic strategy. Int J Cardiol.

[55] Sullivan SD, Mauskopf JA, Augustovski F, Jaime Caro J, Lee KM, Minchin M, Orlewska E, Penna P, Rodriguez Barrios JM, Shau WY (2014). Budget impact analysis - principles of good practice: report of the ISPOR 2012 budget impact analysis good practice II task force. Value Health.

[56] Simeone RM, Oster ME, Cassell CH, Armour BS, Gray DT, Honein MA (2014). Pediatric inpatient hospital resource use for congenital heart defects. Birth Defects Res A Clin Mol Teratol.

[57] Petersen S, Hagglof B, Stenlund H, Bergstrom E (2009). Psychometric properties of the Swedish PedsQL, pediatric quality of life inventory 4.0 generic core scales. Acta Paediatr.

[58] Ranganathan P, Pramesh CS, Buyse M (2016). Common pitfalls in statistical analysis: the perils of multiple testing. Perspect Clin Res.

[59] Berben L, Sereika SM, Engberg S (2012). Effect size estimation: methods and examples. Int J Nurs Stud.

[60] Acuna Mora M, Saarijarvi M, Moons P, Sparud-Lundin C, Bratt EL, Goossens E (2019). The scope of research on transfer and transition in young persons with chronic conditions. J Adolesc Health.

[61] Bodell A, Björkhem G, Thilén U, Naumburg E (2017). National quality register of congenital heart diseases–can we trust the data?. J Congenital Cardiol.

[62] Wulf F, Krasuska M, Bullinger M. Determinants of decision-making and patient participation in paediatric clinical trials: A literature review. Open J Pediatr. 2012;2(1).

[63] Sheridan R, Martin-Kerry J, Hudson J, Parker A, Bower P, Knapp PJT (2020). Why do patients take part in research?. Overview Syst Rev Psychosoc Barriers Facilit.

