# Supplementary material for: Factors affecting adolescents’ participation in randomized controlled trials evaluating the effectiveness of healthcare interventions: the case of the STEPSTONES project
Source: BMC Med Res Methodol. 2020 Aug 3;20:205. doi: 10.1186/s12874-020-01088-7 (PMC7398069; doi:10.1186/s12874-020-01088-7)
Supplement: Supplementary file 1 — Additional file 1. Good Reporting of A Mixed Methods Study (GRAMMS) Checklist. [file 12874_2020_1088_MOESM1_ESM.docx]

**Additional file 1**

**Good Reporting of A Mixed Methods Study (GRAMMS) Checklist**

| **Guideline** | **Section: page** |
| --- | --- |
| Describe the justification for using a mixed methods approach to the research question | Methods: design, p. 2-3 |
| Describe the design in terms of purpose, priority and sequence of methods | Methods: design, p. 2-3 |
| Describe each method in terms of sampling, data collection and analysis | Methods: sample, p. 4-6 |
| Describe where integration has occurred, how it occurred and who participated in it | Discussion: p. 11 |
| Describe any limitation of one method associated with the present of the other method | N/A |
| Describe any insights gained from mixing or integrating methods | Discussion: p. 13 |

**Reference:**

O'Cathain A, Murphy E, Nicholl J. The quality of mixed methods studies in health services research. *J Health Serv Res Policy*. 2008;13: 92-98.
