# Supplementary material for: Factors affecting adolescents’ participation in randomized controlled trials evaluating the effectiveness of healthcare interventions: the case of the STEPSTONES project
Source: BMC Med Res Methodol. 2020 Aug 3;20:205. doi: 10.1186/s12874-020-01088-7 (PMC7398069; doi:10.1186/s12874-020-01088-7)
Supplement: Supplementary file 2 — Additional file 2. Interview guide for the qualitative study. [file 12874_2020_1088_MOESM2_ESM.docx]

**Additional file 2**

**Interview guide for the qualitative study**

| **Research question** | **Interview question** | **Follow up questions/probing questions** |
| --- | --- | --- |
| *What were the adolescent’s own reasons for participating in the* ***research study*?*** | Can you tell me why you agreed to participate in the study? | Why do you think so?  Could you describe more in detail what was the reason for you making this decision?  Was it your own, your parents, or anyone else’s decision? |
| *What factors affected the adolescent decision in participating in the study?* | What do you think are the benefits of participating in this kind of study? | Could you describe more in detail?  So, by saying this, you mean…? |
| *What factors affects adolescents in not participating in these types of research studies?* | What do you think are reasons for not wanting to participate in this kind of study? | Why do you think so?  Could you give me any examples? |

*In the beginning of the interview, we clarified for the interviewee that *research study,* in this context, meant the STEPSTONES randomized controlled trial evaluating the effectiveness of a transition program for adolescents with congenital heart disease.
